# 2-(5-Fluoro-3-isopropyl­sulfanyl-7-methyl-1-benzofuran-2-yl)acetic acid

**DOI:** 10.1107/S1600536812008240

**Published:** 2012-03-03

**Authors:** Hong Dae Choi, Pil Ja Seo, Uk Lee

**Affiliations:** a Department of Chemistry, Dongeui University, San 24 Kaya-dong Busanjin-gu, Busan 614-714, Republic of Korea; b Department of Chemistry, Pukyong National University, 599-1 Daeyeon 3-dong, Nam-gu, Busan 608-737, Republic of Korea

## Abstract

The title compound, C_14_H_15_FO_3_S, was prepared by alkaline hydrolysis of ethyl 2-(5-fluoro-3-isopropyl­sulfanyl-7-methyl-1-benzofuran-2-yl)acetate. In the crystal, mol­ecules are linked *via* pairs of O—H⋯O hydrogen bonds, forming inversion dimers. These dimers are connected by weak C—H⋯O hydrogen bonds.

## Related literature

For background information and the crystal structures of related compounds, see: Seo *et al.* (2011 ▶, 2012 ▶).
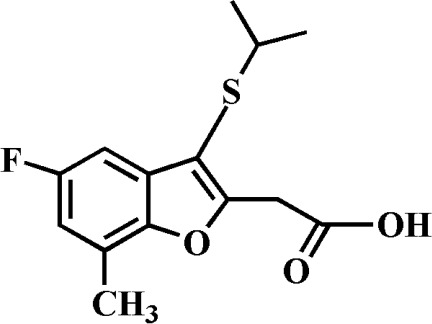


## Experimental

### 

#### Crystal data

C_14_H_15_FO_3_S
*M* *_r_* = 282.32
Triclinic, 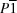

*a* = 8.4365 (2) Å
*b* = 9.2771 (2) Å
*c* = 9.7956 (2) Å
α = 91.404 (1)°
β = 91.2710 (1)°
γ = 115.111 (1)°
*V* = 693.55 (3) Å^3^
*Z* = 2
Mo *K*α radiation
μ = 0.25 mm^−1^
*T* = 173 K
0.35 × 0.32 × 0.28 mm

#### Data collection

Bruker SMART APEXII CCD diffractometer
Absorption correction: multi-scan (*SADABS*; Bruker, 2009 ▶) *T*_min_ = 0.919, *T*_max_ = 0.934
12921 measured reflections
3467 independent reflections
2946 reflections with *I* > 2σ(*I*)
*R*_int_ = 0.028

#### Refinement

*R*[*F* ^2^ > 2σ(*F* ^2^)] = 0.051
*wR*(*F* ^2^) = 0.155
*S* = 1.06
3467 reflections
179 parameters
H atoms treated by a mixture of independent and constrained refinement
Δρ_max_ = 0.93 e Å^−3^
Δρ_min_ = −0.44 e Å^−3^

### 

Data collection: *APEX2* (Bruker, 2009 ▶); cell refinement: *SAINT* (Bruker, 2009 ▶); data reduction: *SAINT*; program(s) used to solve structure: *SHELXS97* (Sheldrick, 2008 ▶); program(s) used to refine structure: *SHELXL97* (Sheldrick, 2008 ▶); molecular graphics: *ORTEP-3* (Farrugia, 1997 ▶) and *DIAMOND* (Brandenburg, 1998 ▶); software used to prepare material for publication: *SHELXL97*.

## Supplementary Material

Crystal structure: contains datablock(s) global, I. DOI: 10.1107/S1600536812008240/ld2050sup1.cif

Structure factors: contains datablock(s) I. DOI: 10.1107/S1600536812008240/ld2050Isup2.hkl

Supplementary material file. DOI: 10.1107/S1600536812008240/ld2050Isup3.cml

Additional supplementary materials:  crystallographic information; 3D view; checkCIF report

## Figures and Tables

**Table 1:** Hydrogen-bond geometry (Å, °)

*D*—H⋯*A*	*D*—H	H⋯*A*	*D*⋯*A*	*D*—H⋯*A*
O3—H3*O*⋯O2^i^	0.86 (4)	1.76 (4)	2.616 (2)	176 (4)
C9—H9*B*⋯O3^ii^	0.98	2.56	3.534 (3)	171

Symmetry codes: (i) 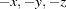; (ii) 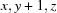.

**Fig. 1.**
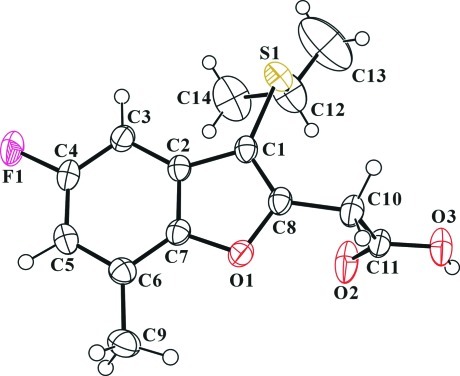
The molecular structure of the title compound with the atom numbering scheme. Displacement ellipsoids are drawn at the 50% probability level. H atoms are presented as small spheres of arbitrary radius.

**Fig. 2.**
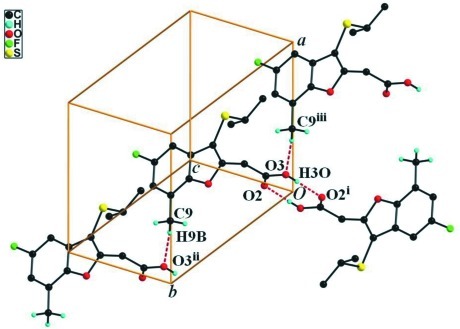
A view of the O–H···O and C–H···O interactions (dotted lines) in the crystal structure of the title compound. H atoms non-participating in hydrogen-bonding were omitted for clarity. [Symmetry codes: (i) - x, - y, - z; (ii) x, y + 1, z; (iii) x, y - 1, z.]

## supplementary crystallographic information

### Comment

As a part of our continuing study of 2-(5-halo-3-isopropylsulfanyl-1-benzofuran-2-yl)acetic acid derivatives containing 5-fluoro (Seo *et al.* , 2011) and 5-bromo (Seo *et al.* , 2012) substituents, we report herein the crystal structure of the title compound.

In the title molecule (Fig. 1), the benzofuran unit is essentially planar, with a mean deviation of 0.010 (1) Å from the least-squares plane defined by the nine constituent atoms. In the crystal structure, the carboxyl groups are involved in intermolecular O–H···O hydrogen bonds (Fig. 2 & Table 1), which link the molecules into centrosymmetric dimers. These dimers are stuck by weak intermolecular C–H···O hydrogen bonds (Fig. 2 & Table 1).

### Experimental

Ethyl 2-(5-fluoro-3-isopropylsulfanyl-7-methyl-1-benzofuran-2-yl)acetate (372 mg, 1.2 mmol) was added to a solution of potassium hydroxide (336 mg, 6 mmol) in water (10 ml) and methanol (10 ml), and the mixture was refluxed for 6h, then cooled. Water was added, and the solution was extracted with dichloromethane. The aqueous layer was acidified to pH=1 with concentrated hydrochloric acid and then extracted with chloroform. The organic layer was separated, dried over magnesium sulfate, filtered and concentrated at reduced pressure. The residue was purified by column chromatography (ethyl acetate) to afford the title compound as a colorless solid [yield 87%, m.p. 436-437 K; *R*_f_ = 0.44 (ethyl acetate)]. Single crystals suitable for X-ray diffraction were prepared by slow evaporation of a solution of the title compound in diisopropyl ether at room temperature.

### Refinement

H atom in the carboxyl group is found in a different Fourier map and refined freely. The other H atoms of C atoms were positioned geometrically and refined using a riding model, with C–H = 0.95 Å for aryl, 1.0 Å for methine, 0.99 Å for methylene and 0.98 Å for methyl H atoms, respectively. *U*_iso_(H) =1.2*U*_eq_(C) for aryl, methine, and methylene, and 1.5*U*_eq_(C) for methyl H atoms. The positions of methyl hydrogens were optimized rotationally. The split of C13 atom was ignored because the disordered ellipsoids also split into two.

### Crystal data

**Table:**

C_14_H_15_FO_3_S	*Z* = 2
*M**_r_* = 282.32	*F*(000) = 296
Triclinic, *P*1	*D*_x_ = 1.352 Mg m^−^^3^
Hall symbol: -P 1	Mo *K*α radiation, λ = 0.71073 Å
*a* = 8.4365 (2) Å	Cell parameters from 5886 reflections
*b* = 9.2771 (2) Å	θ = 2.4–28.4°
*c* = 9.7956 (2) Å	µ = 0.25 mm^−^^1^
α = 91.404 (1)°	*T* = 173 K
β = 91.2710 (1)°	Block, colourless
γ = 115.111 (1)°	0.35 × 0.32 × 0.28 mm
*V* = 693.55 (3) Å^3^	

### Data collection

**Table:**

Bruker SMART APEXII CCD diffractometer	3467 independent reflections
Radiation source: rotating anode	2946 reflections with *I* > 2σ(*I*)
Graphite multilayer monochromator	*R*_int_ = 0.028
Detector resolution: 10.0 pixels mm^-1^	θ_max_ = 28.4°, θ_min_ = 2.1°
φ and ω scans	*h* = −11→11
Absorption correction: multi-scan (*SADABS*; Bruker, 2009)	*k* = −11→12
*T*_min_ = 0.919, *T*_max_ = 0.934	*l* = −13→13
12921 measured reflections	

### Refinement

**Table:**

Refinement on *F*^2^	Primary atom site location: structure-invariant direct methods
Least-squares matrix: full	Secondary atom site location: difference Fourier map
*R*[*F*^2^ > 2σ(*F*^2^)] = 0.051	Hydrogen site location: difference Fourier map
*wR*(*F*^2^) = 0.155	H atoms treated by a mixture of independent and constrained refinement
*S* = 1.06	*w* = 1/[σ^2^(*F*_o_^2^) + (0.0853*P*)^2^ + 0.3723*P*] where *P* = (*F*_o_^2^ + 2*F*_c_^2^)/3
3467 reflections	(Δ/σ)_max_ < 0.001
179 parameters	Δρ_max_ = 0.93 e Å^−^^3^
0 restraints	Δρ_min_ = −0.44 e Å^−^^3^

### Special details

**Table:**

Geometry. All esds (except the esd in the dihedral angle between two l.s. planes) are estimated using the full covariance matrix. The cell esds are taken into account individually in the estimation of esds in distances, angles and torsion angles; correlations between esds in cell parameters are only used when they are defined by crystal symmetry. An approximate (isotropic) treatment of cell esds is used for estimating esds involving l.s. planes.
Refinement. Refinement of F^2^ against ALL reflections. The weighted R-factor wR and goodness of fit S are based on F^2^, conventional R-factors R are based on F, with F set to zero for negative F^2^. The threshold expression of F^2^ > 2sigma(F^2^) is used only for calculating R-factors(gt) etc. and is not relevant to the choice of reflections for refinement. R-factors based on F^2^ are statistically about twice as large as those based on F, and R- factors based on ALL data will be even larger.

### Fractional atomic coordinates and isotropic or equivalent isotropic displacement parameters (Å^2^)

**Table:**

	*x*	*y*	*z*	*U*_iso_*/*U*_eq_	
S1	0.64151 (7)	0.31790 (6)	0.28518 (5)	0.03564 (17)	
F1	0.78061 (17)	0.98655 (14)	0.35845 (16)	0.0503 (4)	
O1	0.23013 (17)	0.40794 (15)	0.34150 (13)	0.0310 (3)	
O2	0.1261 (3)	0.16845 (17)	0.07988 (16)	0.0552 (5)	
O3	0.0331 (2)	−0.07438 (16)	0.15984 (16)	0.0434 (4)	
H3O	−0.022 (5)	−0.102 (5)	0.082 (4)	0.097 (13)*	
C1	0.4901 (2)	0.3980 (2)	0.30894 (17)	0.0273 (4)	
C2	0.5220 (2)	0.5633 (2)	0.32776 (16)	0.0261 (3)	
C3	0.6709 (2)	0.7080 (2)	0.33113 (19)	0.0309 (4)	
H3	0.7852	0.7141	0.3203	0.037*	
C4	0.6403 (3)	0.8411 (2)	0.3512 (2)	0.0343 (4)	
C5	0.4765 (3)	0.8394 (2)	0.3669 (2)	0.0348 (4)	
H5	0.4664	0.9371	0.3791	0.042*	
C6	0.3272 (3)	0.6965 (2)	0.36477 (18)	0.0312 (4)	
C7	0.3583 (2)	0.5618 (2)	0.34579 (17)	0.0273 (4)	
C8	0.3153 (2)	0.3132 (2)	0.31893 (17)	0.0287 (4)	
C9	0.1464 (3)	0.6865 (3)	0.3815 (2)	0.0426 (5)	
H9A	0.0604	0.5752	0.3705	0.064*	
H9B	0.1222	0.7497	0.3122	0.064*	
H9C	0.1386	0.7283	0.4728	0.064*	
C10	0.2049 (3)	0.1387 (2)	0.30994 (19)	0.0349 (4)	
H10A	0.1139	0.1115	0.3792	0.042*	
H10B	0.2789	0.0828	0.3322	0.042*	
C11	0.1170 (3)	0.0792 (2)	0.17141 (19)	0.0317 (4)	
C12	0.6510 (5)	0.3118 (4)	0.0971 (3)	0.0624 (8)	
H12	0.5284	0.2565	0.0581	0.075*	
C13	0.7450 (8)	0.2152 (6)	0.0605 (5)	0.124 (2)	
H13A	0.7367	0.1960	−0.0388	0.186*	
H13B	0.6925	0.1132	0.1055	0.186*	
H13C	0.8683	0.2722	0.0904	0.186*	
C14	0.7333 (5)	0.4753 (4)	0.0408 (3)	0.0766 (10)	
H14A	0.8535	0.5318	0.0783	0.115*	
H14B	0.6652	0.5344	0.0663	0.115*	
H14C	0.7351	0.4665	−0.0590	0.115*	

### Atomic displacement parameters (Å^2^)

**Table:**

	*U*^11^	*U*^22^	*U*^33^	*U*^12^	*U*^13^	*U*^23^
S1	0.0454 (3)	0.0356 (3)	0.0327 (3)	0.0233 (2)	0.0074 (2)	0.00516 (18)
F1	0.0421 (7)	0.0235 (6)	0.0731 (9)	0.0025 (5)	0.0028 (6)	−0.0043 (6)
O1	0.0274 (6)	0.0274 (6)	0.0340 (7)	0.0077 (5)	−0.0037 (5)	0.0021 (5)
O2	0.0785 (12)	0.0260 (7)	0.0413 (8)	0.0041 (7)	−0.0244 (8)	0.0048 (6)
O3	0.0606 (10)	0.0224 (6)	0.0362 (8)	0.0077 (6)	−0.0109 (7)	0.0002 (5)
C1	0.0329 (9)	0.0237 (8)	0.0232 (7)	0.0103 (7)	−0.0018 (6)	−0.0003 (6)
C2	0.0304 (9)	0.0243 (8)	0.0217 (7)	0.0101 (7)	−0.0009 (6)	−0.0001 (6)
C3	0.0278 (9)	0.0277 (8)	0.0334 (9)	0.0082 (7)	0.0014 (7)	−0.0006 (7)
C4	0.0365 (10)	0.0232 (8)	0.0354 (9)	0.0056 (7)	−0.0011 (7)	−0.0015 (7)
C5	0.0436 (11)	0.0269 (8)	0.0353 (9)	0.0164 (8)	0.0006 (8)	−0.0004 (7)
C6	0.0342 (10)	0.0329 (9)	0.0287 (8)	0.0164 (8)	−0.0014 (7)	0.0023 (7)
C7	0.0285 (8)	0.0261 (8)	0.0244 (8)	0.0091 (7)	−0.0034 (6)	0.0013 (6)
C8	0.0324 (9)	0.0243 (8)	0.0259 (8)	0.0088 (7)	−0.0038 (6)	0.0012 (6)
C9	0.0393 (11)	0.0434 (11)	0.0521 (12)	0.0240 (9)	0.0007 (9)	0.0072 (9)
C10	0.0392 (10)	0.0245 (8)	0.0326 (9)	0.0057 (7)	−0.0070 (8)	0.0034 (7)
C11	0.0328 (9)	0.0229 (8)	0.0332 (9)	0.0061 (7)	−0.0037 (7)	0.0015 (6)
C12	0.097 (2)	0.0646 (16)	0.0406 (12)	0.0479 (16)	0.0232 (13)	0.0050 (11)
C13	0.202 (6)	0.151 (4)	0.086 (3)	0.135 (5)	0.063 (3)	0.026 (3)
C14	0.116 (3)	0.076 (2)	0.0474 (15)	0.048 (2)	0.0261 (16)	0.0178 (14)

### Geometric parameters (Å, º)

**Table:**

S1—C1	1.7464 (19)	C6—C9	1.502 (3)
S1—C12	1.847 (3)	C8—C10	1.485 (2)
F1—C4	1.365 (2)	C9—H9A	0.9800
O1—C8	1.368 (2)	C9—H9B	0.9800
O1—C7	1.376 (2)	C9—H9C	0.9800
O2—C11	1.217 (2)	C10—C11	1.506 (3)
O3—C11	1.295 (2)	C10—H10A	0.9900
O3—H3O	0.86 (4)	C10—H10B	0.9900
C1—C8	1.352 (3)	C12—C13	1.469 (4)
C1—C2	1.446 (2)	C12—C14	1.501 (4)
C2—C7	1.390 (3)	C12—H12	1.0000
C2—C3	1.395 (2)	C13—H13A	0.9800
C3—C4	1.375 (3)	C13—H13B	0.9800
C3—H3	0.9500	C13—H13C	0.9800
C4—C5	1.387 (3)	C14—H14A	0.9800
C5—C6	1.387 (3)	C14—H14B	0.9800
C5—H5	0.9500	C14—H14C	0.9800
C6—C7	1.390 (3)		
			
C1—S1—C12	101.58 (11)	C6—C9—H9C	109.5
C8—O1—C7	105.60 (14)	H9A—C9—H9C	109.5
C11—O3—H3O	110 (3)	H9B—C9—H9C	109.5
C8—C1—C2	105.76 (16)	C8—C10—C11	113.50 (15)
C8—C1—S1	125.54 (14)	C8—C10—H10A	108.9
C2—C1—S1	128.66 (14)	C11—C10—H10A	108.9
C7—C2—C3	119.79 (16)	C8—C10—H10B	108.9
C7—C2—C1	105.53 (15)	C11—C10—H10B	108.9
C3—C2—C1	134.68 (17)	H10A—C10—H10B	107.7
C4—C3—C2	115.14 (17)	O2—C11—O3	123.96 (17)
C4—C3—H3	122.4	O2—C11—C10	122.48 (16)
C2—C3—H3	122.4	O3—C11—C10	113.55 (16)
F1—C4—C3	118.24 (18)	C13—C12—C14	112.4 (3)
F1—C4—C5	116.81 (17)	C13—C12—S1	107.5 (2)
C3—C4—C5	124.94 (17)	C14—C12—S1	112.2 (2)
C4—C5—C6	120.63 (17)	C13—C12—H12	108.2
C4—C5—H5	119.7	C14—C12—H12	108.2
C6—C5—H5	119.7	S1—C12—H12	108.2
C5—C6—C7	114.44 (17)	C12—C13—H13A	109.5
C5—C6—C9	123.24 (18)	C12—C13—H13B	109.5
C7—C6—C9	122.32 (18)	H13A—C13—H13B	109.5
O1—C7—C2	110.54 (15)	C12—C13—H13C	109.5
O1—C7—C6	124.41 (17)	H13A—C13—H13C	109.5
C2—C7—C6	125.04 (17)	H13B—C13—H13C	109.5
C1—C8—O1	112.56 (15)	C12—C14—H14A	109.5
C1—C8—C10	130.95 (18)	C12—C14—H14B	109.5
O1—C8—C10	116.49 (16)	H14A—C14—H14B	109.5
C6—C9—H9A	109.5	C12—C14—H14C	109.5
C6—C9—H9B	109.5	H14A—C14—H14C	109.5
H9A—C9—H9B	109.5	H14B—C14—H14C	109.5
			
C12—S1—C1—C8	91.91 (19)	C3—C2—C7—C6	1.5 (3)
C12—S1—C1—C2	−91.00 (18)	C1—C2—C7—C6	−178.68 (16)
C8—C1—C2—C7	−0.86 (19)	C5—C6—C7—O1	179.41 (16)
S1—C1—C2—C7	−178.39 (13)	C9—C6—C7—O1	−0.6 (3)
C8—C1—C2—C3	178.94 (19)	C5—C6—C7—C2	−1.0 (3)
S1—C1—C2—C3	1.4 (3)	C9—C6—C7—C2	179.04 (18)
C7—C2—C3—C4	−0.7 (3)	C2—C1—C8—O1	0.5 (2)
C1—C2—C3—C4	179.49 (18)	S1—C1—C8—O1	178.10 (12)
C2—C3—C4—F1	178.68 (16)	C2—C1—C8—C10	−179.33 (17)
C2—C3—C4—C5	−0.4 (3)	S1—C1—C8—C10	−1.7 (3)
F1—C4—C5—C6	−178.20 (17)	C7—O1—C8—C1	0.13 (19)
C3—C4—C5—C6	0.9 (3)	C7—O1—C8—C10	179.95 (14)
C4—C5—C6—C7	−0.2 (3)	C1—C8—C10—C11	−98.2 (2)
C4—C5—C6—C9	179.80 (19)	O1—C8—C10—C11	82.0 (2)
C8—O1—C7—C2	−0.71 (18)	C8—C10—C11—O2	−5.3 (3)
C8—O1—C7—C6	178.95 (16)	C8—C10—C11—O3	174.37 (18)
C3—C2—C7—O1	−178.85 (15)	C1—S1—C12—C13	−168.1 (3)
C1—C2—C7—O1	0.98 (19)	C1—S1—C12—C14	67.9 (3)

### Hydrogen-bond geometry (Å, º)

**Table:**

*D*—H···*A*	*D*—H	H···*A*	*D*···*A*	*D*—H···*A*
O3—H3*O*···O2^i^	0.86 (4)	1.76 (4)	2.616 (2)	176 (4)
C9—H9*B*···O3^ii^	0.98	2.56	3.534 (3)	171

Symmetry codes: (i) −*x*, −*y*, −*z*; (ii) *x*, *y*+1, *z*.
